# Automated identification of blastocyst regions at different development stages

**DOI:** 10.1038/s41598-022-26386-6

**Published:** 2023-01-02

**Authors:** Adolfo Flores-Saiffe Farias, Alejandro Chavez-Badiola, Gerardo Mendizabal-Ruiz, Roberto Valencia-Murillo, Andrew Drakeley, Jacques Cohen, Elizabeth Cardenas-Esparza

**Affiliations:** 1 IVF 2.0 Limited, Merseyside, UK; 2 New Hope Fertility Center, Mexico City, Mexico; 3 Reproductive Genetics, School of Biosciences, Univeresity of Kent, Kent, UK; 4 Departamento de Ciencias Computacionales, Universidad de Guadalajara, Guadalajara, Mexico; 5 Hewitt Centre for Reproductive Medicine, Liverpool Women’s Hospital, Liverpool, UK; 6 IVFqc, Hudson, New York USA; 7 New Hope Fertility Center, Guadalajara, Mexico

**Keywords:** Computational models, Image processing, Machine learning, Embryology, Pattern formation, Computer science

## Abstract

The selection of the best single blastocyst for transfer is typically based on the assessment of the morphological characteristics of the zona pellucida (ZP), trophectoderm (TE), blastocoel (BC), and inner cell-mass (ICM), using subjective and observer-dependent grading protocols. We propose the first automatic method for segmenting all morphological structures during the different developmental stages of the blastocyst (i.e., expansion, hatching, and hatched). Our database contains 592 original raw images that were augmented to 2132 for training and 55 for validation. The mean Dice similarity coefficient (DSC) was 0.87 for all pixels, and for the BC, BG (background), ICM, TE, and ZP was 0.85, 0.96, 0.54, 0.63, and 0.71, respectively. Additionally, we tested our method against a public repository of 249 images resulting in accuracies of 0.96 and 0.93 and DSC of 0.67 and 0.67 for ICM and TE, respectively. A sensitivity analysis demonstrated that our method is robust, especially for the BC, BG, TE, and ZP. It is concluded that our approach can automatically segment blastocysts from different laboratory settings and developmental phases of the blastocysts, all within a single pipeline. This approach could increase the knowledge base for embryo selection.

## Introduction

In-vitro fertilization (IVF) is one of the most common and effective methods for the treatment of infertility. This procedure consists of stimulating a woman’s ovaries to generate multiple eggs in a given cycle. The mature eggs are retrieved and placed in a Petri dish where they are fertilized by sperm and cultured in an incubator under controlled environmental conditions^1^. In the days to follow, successfully fertilized eggs, now embryos, will undergo different stages of development until after five to six days some will reach the blastocyst stage. A blastocyst may then be transferred to the woman’s uterus with the intention of generating a pregnancy.

**Figure 1:**
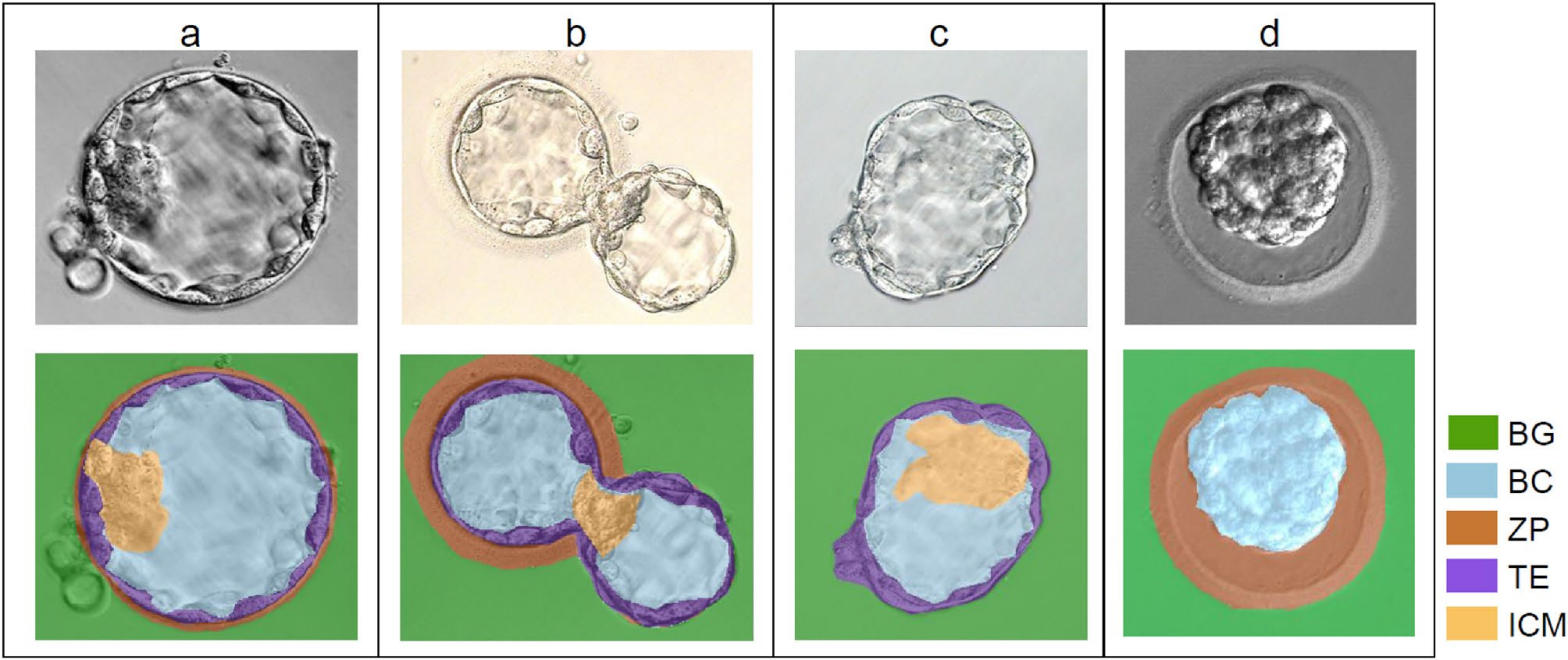
Examples of the blastocycst structures at each developmental stage, expansion (**a**), hatching (**b**), hatched (**c**) and collapsed (**d**). Colours indicate the regions of interest in the micrograph: background(BG), blastocoel (BC), zona pellucida (ZP), trophectoderm (TE), and the embryoblast or inner cell mass (ICM).

The blastocyst is the first morphologically differentiated state of the human pre-implantation embryo, in which cellular structures are arranged in at least four regions: the trophectoderm (TE), which is a layer of cells that surrounds a fluid cavity known as the blastocoel (BC), the embryoblast or inner cell mass (ICM), and the zona pellucida (ZP), which is a protective layer. The development stage of the blastocyst also might be defined by at least three phases: expansion and thinning of the zona pellucida, hatching of the embryo through the zona pellucida, and hatched from the zona pellucida referring to the process of leaving the ZP. These stages are part of the criteria used by embryologists when evaluating an embryo’s quality^2^. Figure 1 depicts the structures of expansion, hatching and hatched blastocysts.

In many IVF treatments, multiple blastocysts are available for transfer. While transferring multiple blastocysts is sometimes considered as an option to increase the chances of a successful pregnancy, this practice is not recommended due to the possibility of a multiple pregnancy and its associated risks including premature birth, the need for a cesarean section, and a higher risk for pregnancy loss, and other maternal and neonatal morbidities. In that context, a single embryo transfer is a better alternative, yet this necessitates improving embryo classification.

The selection of the best blastocyst for transfer is commonly based on the assessment of morphological characteristics and rate of development, although there are more invasive and expensive approaches, including embryo biopsy and genetic testing, that require cryopreservation. The most common way to perform morphological assessment has been by visual inspection of the embryos or using a digital image source. The assignment of quality scores, such as those proposed by Dokras and Gardner, have been shown to be advantageous^3,4^, but a major limitation of this approach is subjectivity, which is related to judgement, training, and expertise.

Automatic identification of the discrete regions of a blastocyst using digital microscopy images could aid in overcoming the drawbacks of the historical methods, by increasing the objectivity and reproducibility of the embryo selection process. These computer-based analysis tools could provide valuable quantitative information to the embryologist to support and improve the decision-making process during an IVF treatment. For instance, based on the intensity patterns of the regions in the image of a blastocyst, it is possible to automatically infer the embryo quality or its potential by using artificial intelligence (AI)^5,6^.

Current work related to the automatic, regional segmentation of blastocysts from microscopy images can be divided into two categories. The first category corresponds to methods that rely on the use of computer vision filters and segmentation methods such as watershed segmentation^7–9^ or by the use of parametric curves such as ellipses^10^, active contour models^11^, and level sets^12,13^. The second type of methods are those based on the use of supervised machine learning classifiers that are capable of predicting a class-label to each pixel of the image according to the pattern contained in a feature vector that is generated using computer vision filters^14–16^. Most recent methods in this category rely on the use of deep convolutional neural networks (CNN) to automatically determine the best features to extract from the image and to perform the segmentation by defining a label to each pixel using encoder-decoder architectures^17–23^.

Despite the existence of these methods, the automatic segmentation of the blastocyst continues to be a challenging task, due to the large variability of shapes and image intensity patterns of each blastocyst region during the different stages of late embryo development, including the expansion, hatching, and hatched stages (See Fig. 1). Furthermore, most of the reports in the literature focus on performing the segmentation of a single region of the blastocyst (e.g., ICM^8,11,17,18^, TE^13,23^, ZP^10,16^), and only some are designed to perform the simultaneous segmentation of two (e.g., TE and ICM^7,19^), three (e.g., ZP, TE, ICM^12^) or four (e.g.,^14,21,22^) regions of interest using blastocyst micrographs. Additionally and to the best of our knowledge, only two works^9,20^ have included hatching embryos, but they only segmented the background from the embryo, and only one work^9^ included images from different laboratory settings (e.g. microscopes and cameras, but not magnifications).

In this work we present a method for the fully automatic simultaneous segmentation of the four blastocyst regions (ZP, TE, BC, and ICM) and the BG (culture medium) from digital microscopy images. This method is based on the use of computer vision filters and supervised machine learning classifiers, including deep learning methods. This work differs from previously published work in that our method is capable of segmenting blastocyst images from the later developmental stages (expansion, hatching, and hatched) and from four different laboratory settings (i.e. cameras, magnifications, light conditions, and general laboratory conditions). The performance of the proposed method was evaluated by computing the Dice similarity coefficient (DSC) between the automatic segmentation results and the manual annotations from a senior embryologist. The originality of the work resides in the model’s robustness to adapt to different laboratory settings to segment blastocysts in any phase (i.e. expanding, hatching, hatched) and in the sensitivity analysis of the performance of the pipeline.

## Results

**Figure 2:**
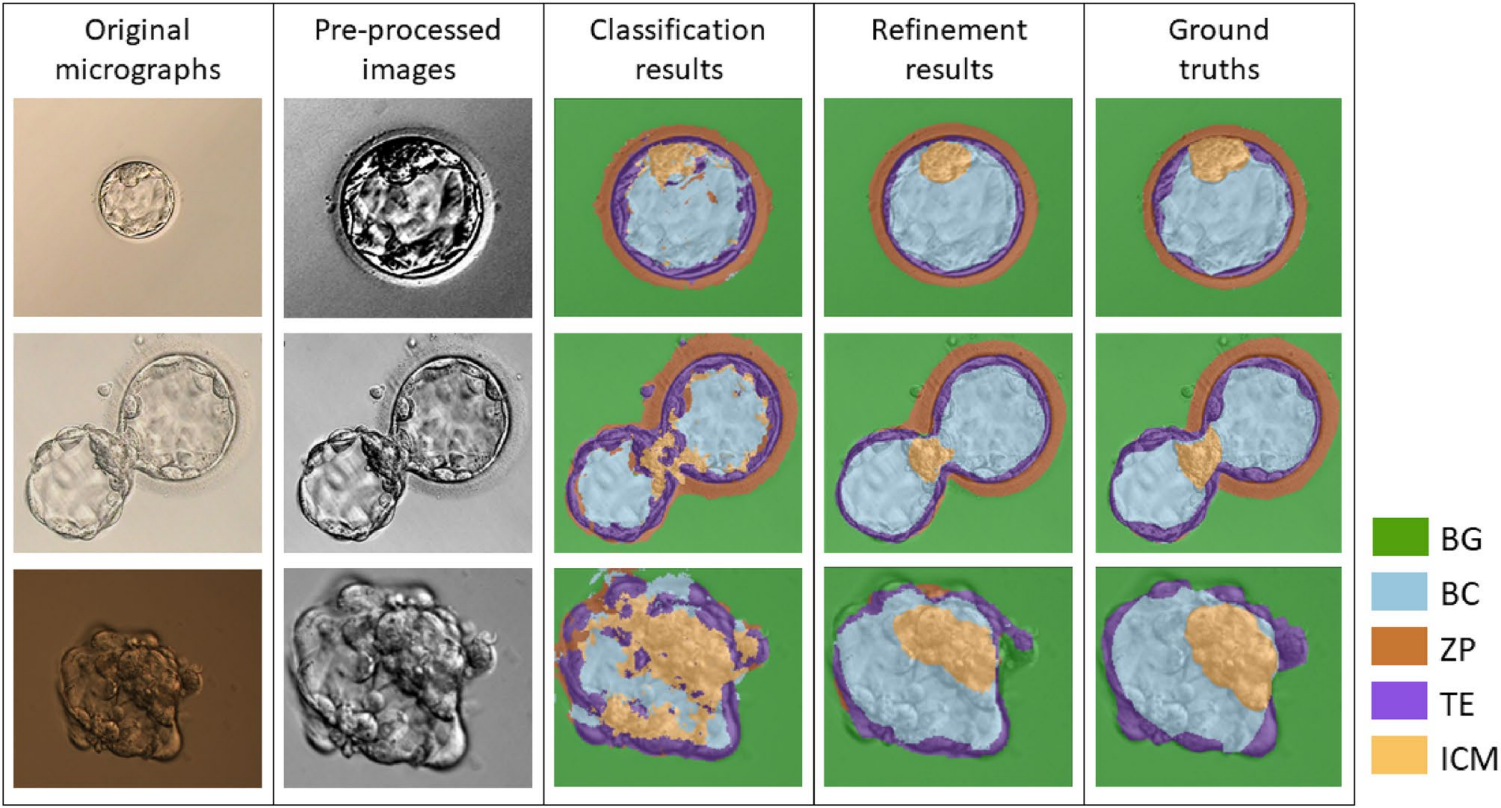
Examples of the original blastocyst micrographs, the images after pre-processing, before and after the segmentation refinement, and the ground truth. Please note that these images might have been stretched, shrunk, or cropped for aesthetic purposes, but the pixel values remained unaltered.

Table 1 lists the DSC results of the proposed segmentation method for each of the regions and each stage, including a subset of only images with ICM ground truth (ICM2) in the augmented testing set. Figure 2 depicts examples of the blastocyst micrographs after pre-processing, and before and after the segmentation refinement along with the ground truth segmentation.

**Table 1:** Mean and standard deviation of Dice similarity coefficient for each region before and after the segmentation refinement step.

Region	Mean (std) DSC before refinement	Mean (std) DSC of the whole dataset	Mean (std) DSC of expansion embryos	DSC of hatching embryos	Mean (std) DSC of hatched embryos
BC	0.72 (0.12)	0.85 (0.06)	0.86 (0.06)	0.84 (0.05)	0.80 (0.11)
BG	0.94 (0.04)	0.96 (0.03)	0.96 (0.02)	0.94 (0.04)	0.95 (0.02)
ICM	0.45 (0.26)	0.54 (0.33)	0.55 (0.32)	0.53 (0.35)	0.44 (0.40)
ICM2*	0.55 (0.18)	0.66 (0.22)	0.66 (0.23)	0.69 (0.21)	0.66 (0.17)
TE	0.59 (0.13)	0.63 (0.13)	0.65 (0.14)	0.58 (0.09)	0.57 (0.08)
ZP	0.66 (0.22)	0.71 (0.24)	0.73 (0.20)	0.75 (0.23)	0.30 (0.51)
All	0.80 (0.06)	0.87 (0.04)	0.88 (0.04)	0.86 (0.05)	0.84 (0.04)

ICM2* - Subset of the embryos filtering those where no ICM was identified by embryologists (20 expansion, and 15 hatching) The ‘All’ row includes the five regions and embryos where no ICM was found.

Note that the segmentation result for all regions improved after the refinement step, with an increment of at least 18% of DSC for the BC and ICM regions. Due to the high homogeneity of texture of the BG and BC regions, the proposed method achieved the best results with a mean DSC above 85%. The ICM segment had the worst DSC followed by TE and ICM2, with high standard deviations. It can be observed in Table 1, that TE was better segmented in expansion embryos than in hatching or hatched embryos. The performance of our model when it was compared to the public database^7^ is shown in the Table 2. This table reports that our procedure is weaker when it is assessed by DSC and sensitivity, but stronger when assessed by specificity, precision, and accuracy.

**Table 2:** Comparison of the results by Saeedi et al., and ours in the same database.

Metric	ICM by saeedi et al.	TE by saeedi et al.	ICM	TE	ZP
DSC	0.79	0.77	0.67	0.67	0.75
Sensitivity	0.84	0.89	0.62	0.59	0.69
Specificity	0.92	0.86	0.99	0.98	0.98
Precision	0.77	0.69	0.87	0.80	0.85
Accuracy	0.91	0.87	0.96	0.93	0.94

*ICM* Inner cell mass, *TE* Trophectoderm, *ZP* Zona pellucida, *DSC* Dice Similarity Coefficient.

**Figure 3:**
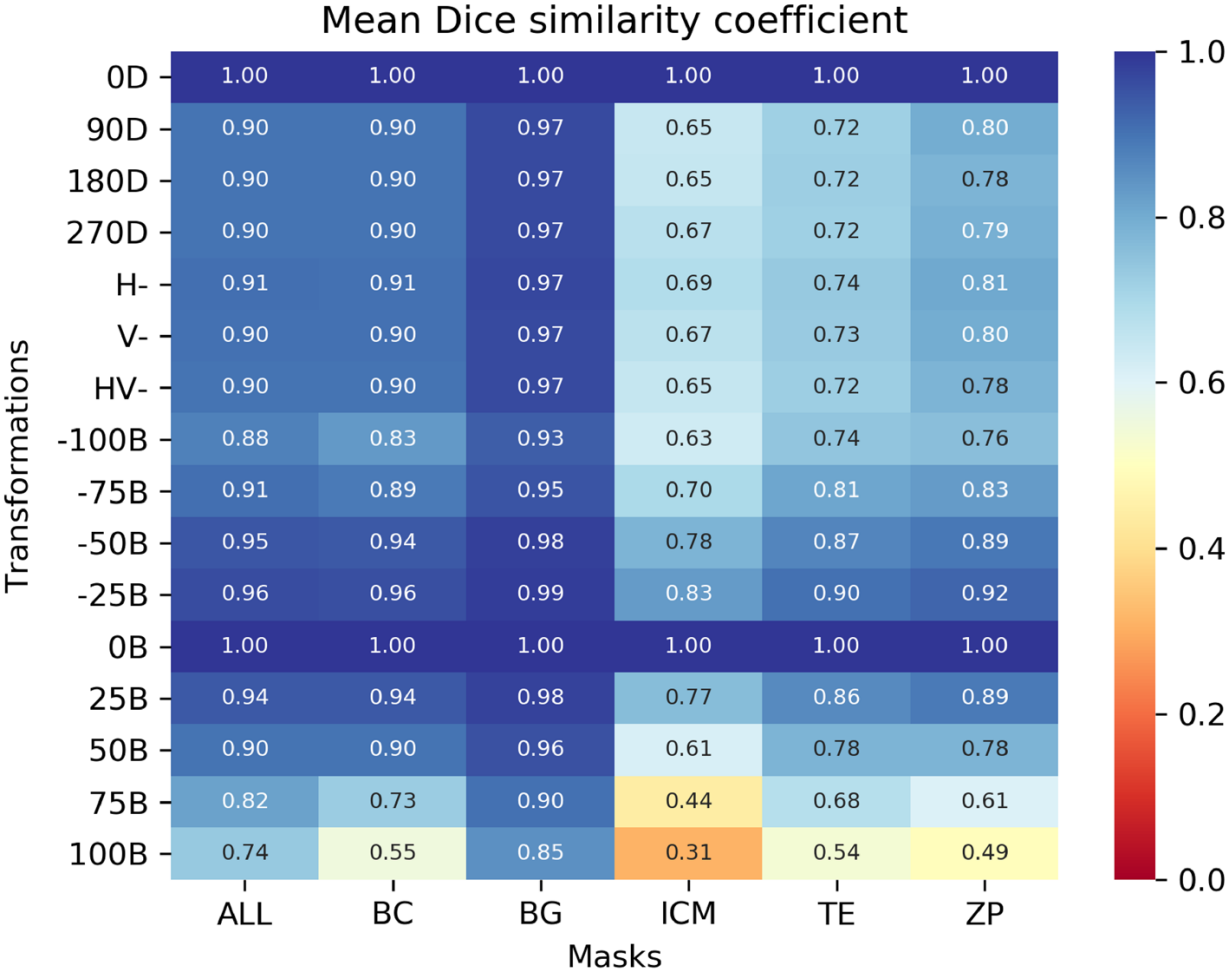
Mean DSC for the regions of interest (x-axis) between the original blastocyst mictrographs and their transformations (y-axis) of the segmentation result. 0D, 90D, 180D, and 270D represents the rotational degrees transformation. H-, V-, and HV- represent horizontal, vertical, or both flip transformations. −100B to 100B represent the absolute value of the brightness transformation. *BC* Blastocoel, *BG* Background, *ICM* Inner cell mass, *TE* trophectoderm, *ZP* zona pellucida, *All* all the regions of interest.

Figure 3 shows the mean DSC of the regions of interest of the transformed blastocyst images compared with the raw images. Note that the ICM segmentation is the most sensitive to the transformations, followed by the TE region, and BG followed by BC are the most robust to transformations. Also, our method is more robust to reduced brightness than increased brightness.

## Discussion

Automatic segmentation of blastocyst regions could potentially overcome drawbacks like inter-/intra-observer variability, and provide novel quantitative tools for blastocyst evaluation, confirming other approaches involving AI-based technologies^5,6^. This work presents a novel method for automatically segmenting the relevant biological structures of blastocysts from micrographs taken at different developmental stages: expansion, hatching, and hatched. We compare our method with two ground truth datasets manually segmented by senior embryologists: (i) 592 blastocyst micrographs that included embryos at the three developmental stages with masks for the BG, ZP, TE, BC, and ICM regions from four different laboratory settings (two clinics and two different magnifications each; dataset developed by our group) and (ii) a public repository of 249 expansion embryos and with masks for ZP, TE, and ICM^7^. The results showed a mean 0.87 DSC across all regions of our dataset (with no relevant difference between blastocyst stages), and a mean 0.79 DSC for the public repository. Additionally, we have performed a sensitivity analysis on an independent database to test the robustness of our method to adapt to different micrographs conditions. We artificially tested different locations and positions of the blastocysts in the micrographs and modified the brightness of the images. However, we understand that there is an inherent limitation and information loss when assessing a 2D image of a 3D structure.

When analysing the performance of this method between different regions, the ZP is highly relevant, since it appears in the micrographs as a thin semi-transparent layer with faint texture that can be difficult to differentiate from the background, especially at its outer limits. Moreover, at the hatching and hatched stages, the ZP could be absent from certain areas of the blastocyst. That might explain why the DSC is near 0.7 since the boundaries of the TE region can be difficult to define. Therefore, although the qualitative results in Fig. 2 appear to be similar to the ground truth, the area overlap may be less, producing a low DSC. Moreover, in the dataset we included 6 highly collapsed embryos where no TE was clearly found by embryologists, which made it a harder problem for the AI.

The ICM was the most challenging region to segment, both for the embryologists, when presented with static images, and for our method. This was perhaps because of its large optical similarity with the TE, or due to those embryos where it could be erroneously confused with TE either because it was out of focus, completely invisible in the image, or the embryo was collapsed. However, the proposed method always tries to find it on the micrographs, and produces a zero DSC when it is absent, which could explain the low mean and high standard deviation. By removing the 107 images with no ICM identified by the embryologists, the score increased from 0.54 to 0.66.

In the second dataset, our method showed higher specificity, and precision, but lower DSC and sensitivity than the results reported by Saeedi et al., in both regions (TE and ICM). These results suggest a high performance among the pixels predicted as TE and ICM (a high true positive rate), but also leaving many pixels among the mask unselected (high false negative rate). This behaviour is demonstrated by nearly absolute specificity (0.99 and 0.98 for ICM and TE respectively), interpreted as correctly identifying almost all the true negative pixels, in contrast with a very low sensitivity (0.62 and 0.59 for ICM and TE respectively). Put simply, when our method classifies a pixel as TE or ICM, it is highly possible that it is true, but there might be many other pixels that our method is missing. However, in the overall accuracy (metric including both true and false positives) our method demonstrated a better performance (0.96 and 0.93 for ICM and TE respectively). When comparing the performance of our method in both datasets, we found a slightly better result in the dataset provided by Saeedi et al., by an increase in DSC of 0.13, 0.04, 0.01, and 0.04 for ICM, ICM2, TE, and ZP. This could be due to the heterogeneity (laboratory settings, blastocysts phases, embryo quality) of our dataset, and the inclusion of collapsed embryos and non-evident ICM, such as embryos photographed while the ICM was not in the focal plane.

Additionally, further work has been done on the same database. Rad et al., in 2018 published a conference paper in which a multi-resolutional ensemble of Stacked Dilated U-Net architecture to segment the ICM from expansion embryos, reporting a precision of 88.6%, recall of 91.5%, accuracy of 98.3%, and SDC of 89.5% on their test set ($n=35$)^18^. The same group a year later reported BLAST-NET, a Dense Progressive Sub-pixel Upsampling architecture to segment all five segments^21^. This architecture showed a Mean SDC of 0.91. Harun et al., in 2019 also reported segmentation performances of ICM and TE (one network for each segment) using a Residual Dilated U-Net. They report that their approach can identify the ICM region with 99.1% accuracy, 94.9% precision, 93.8% recall, and 94.3% Dice Coefficient, and the TE region with 98.3% accuracy, 91.8% precision, 93.2% recall, and 92.5% Dice Coefficient^19^. Despite the performances of these networks apparently overcome ours, it is important to highlight that the database contains several limitations, all the images come from a single laboratory setting of expansion blstocysts only. Our dataset contains 4 different lab-settings, meaning different magnifications, cameras, and light conditions, and also includes embryos at different phases. Our approach also segments five classes within a single model using a two step architecture. Further work would need to be done on different databases and architectures to prove their ability to generalise.

Regarding the sensitivity analysis, we observed the ICM to be the most affected by the different image transformations, followed by TE, ZP and BC. We can also state that increased brightness was more detrimental to the segmentation than reduced brightness. The rotational and flipping transformations had similar detrimental effects across masks, the ICM being the most affected. The sensitivity analysis highlighted the strengths and weaknesses of our segmentation method, providing a path for improving the robustness of the pre-processing and segmentation in later versions. Further studies could be conducted on a larger manually annotated images from different laboratory settings to properly assess the generalizability of the segmentation model.

The Table 1 shows the ability of the autoencoder to refine the segmentation obtained from the texture-based classifier (from 0.80 to 0.87 of DSC for all regions). This novel deep learning architecture shows a hybrid traditional computer vision (top-down) and deep learning approach (bottom-up) of pre-processing and extracting informative features from the images, to further classify each pixel for a second step of refinement including the neighbouring pixels of the segmentation through an encoder-decoder architecture. We foresee that this approach could be applied to other segmentation problems.

Although our method was tested against an external dataset, the generalizability of our model using different protocols, culture methodology, and microscopes remains to be tested. As mentioned above, the blastocysts used for training and validation were developed under a protocol of assisted hatching, which might induce a bias in the performance of the method. Embryos treated with this procedure hatch earlier than untreated embryos, altering important morphological aspects of the blastocyst like the thickness of the ZP, the“figure of eight”shape of the hatching blastocyst, and overall blastocyst size. Other differences in IVF protocols should also be tested, such as culture media, freeze-thawed or biopsied embryos, and many others. Additionally, a limitation of our results was the poor performance in segmenting the ICM, which might be one of the most relevant features for predictive algorithms due to its high biological relevance. It is also fair to assume a bias of our ground truth dataset given the proven existence of inter-/intra-embryologist variability in assessing embryos according to their regions^24^. Finally, the ICM may have to be visualized at different focal positions as the blastocyst is 150-300 micrometres in diameter. Although this is the first work that includes the segmentation of the three late phases of a blastocyst, there is a strong bias due to the data unbalance. Further work must be done in testing different neural network architectures in a phase-balanced database. The limitations of our work could be summarized as having a moderate performance on segmenting ICM, the high phase imbalance in our database, the bias of the blastocysts images being under an assisted hatching protocol, that the manual annotations were performed by a single senior embryologist, and the relatively small database.

While there are methods for blastocyst analysis that do not make use of a segmentation step, the advantage of segmentation is that the data fed to the machine learning models have a structure that depends on the well-identified characteristics of the cell, which can be translated into more transparent and explainable AI models. Potential future clinical applications that might use automated segmentation methods might include blastocyst ploidy status prediction, blastocyst transference outcome, or assistance during TE biopsy.

## Methods

**Figure 4:**
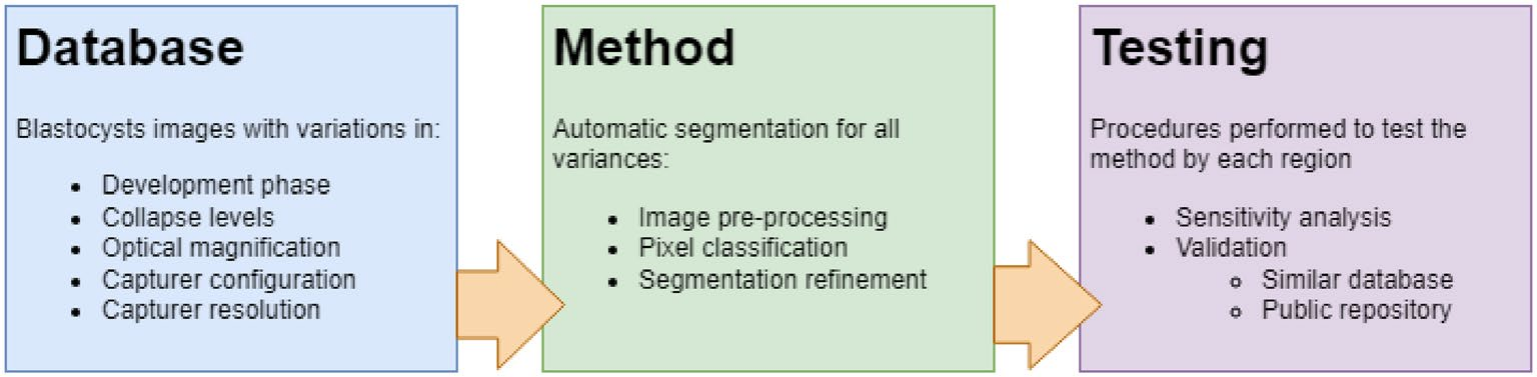
Overview of the materials and methods used in the study.

As an overview, the materials and methods can be summarized into the database description, the segmentation procedure (images pre-processing, pixel classification, and segmentation refinement), and testing the model (sensitivity analysis and validation through a testing set and a public repository) as described in Fig. 4.

### Database and ground truth description

In this work, we employed blastocyst images generated through data augmentation techniques, from a dataset of 592 blastocyst images collected from two IVF clinics during six consecutive months. Informed consent was obtained from all subjects. The image data collection and all experimental protocols were performed in accordance with relevant named guidelines and regulations (IRB approval number RPA-2021-03, IRB name: Comité de Bioética en Investigación New Hope Fertility Center, Registry number: CONBIOETICA 09-CEI-00120170131). The images were collected in two different laboratory settings with the following equipment respectively: two inverted microscopes models IX73 and IX71, (Olympus, Japan), with the capturers LW1135C and DSP3600 series MOD301213 (Hamilton Thorne, Canada), with the magnification objectives LUCplanFLN 20X/40X and LCACHN20X/40X (Olympus, Japan). Embryos were cultured in Continuous Single Culture Complete with HSA (Irvine Scientific, Fujifilm, USA) culture medium. We defined a hatching embryo as a blastocyst with at least one blastocyst cell outside the ZP, and a hatched blastocyst as one with most or all of the blastocyst cells outside the ZP. The database contained images of 372 expansion, 199 hatching, and 21 hatched blastocysts.This unbalance is due to the fact that the laboratory that provided us with the images rarely takes the embryos to the hatched stage, but performs embryo transfer or freeze during expansion or hatching phases. Additionally, the only exclusion criteria is that the focal plane was at the thickest part of the embryo (middle layer), so that the trophectoderm cells can be sharply observed. It was not a requirement that the inner cell mass were clearly observable. It is relevant to note that the clinic performs assisted hatching on most of its embryos (as per internal protocol). This procedure induces the hatching process to occur sooner than for an embryo that has not undergone this procedure. For each image, a senior embryologist defined a bounding box containing the blastocyst with a self-developed software and then performed manual segmentation of the regions of interest (i.e. BG, BC, ICM, TE, and ZP) where possible, also with a self developed software.

In 107 micrographs, the embryologist reported that they were either unsure about the boundaries of the ICM or that it was not visible at all, and they were instructed to not mark any ICM. Also, most of the hatched embryo images lacked a visible ZP.

The result of these procedures is a bounding box for each blastocyst (smallest possible rectangle defined by the upper left and lower right coordinates that contains the whole blastocyst), and a set of masks (binary images where‘1’means that the pixel belongs to the label of the mask and‘0’ otherwise) with the segmented regions where possible.

### Image pre-processing and model training

The method consisted of three steps: (i) pre-processing of the micrographs to segment and standardize the images, (ii) classification of the pixels in the pre-processed image to the relevant blastocyst zone, and (iii) a post-processing refinement to improve the segmentation based on the structure of the blastocyst regions.

Prior to pre-processing, we used random sampling to divide the database into a training set using 90% of the micrographs, and a testing set employing the remaining 10%. Data augmentation techniques were performed on the training set by randomly creating modified versions of each image by variations of brightness (by factors of 0.8, 0.9, 1.0, 1.1 or 1.2), by performing horizontal and vertical flips, and rotations (0, 90, 180, or 270 degrees). After this process, we obtained a training set of 2132 blastocyst images and a testing set of 55 (69% expansion, 24% hatching, and 5% hatched).

#### Image pre-processing

Apart from the embryo of interest, blastocyst micrograph images might contain other cells, or the instruments employed to manipulate them. Also, the blastocyst position on the image might not be centralized. Therefore, the first part of the pre-processing step consists of identifying the minimum region of the micrograph image that contains the blastocyst and excludes any extraneous objects.

**Figure 5:**
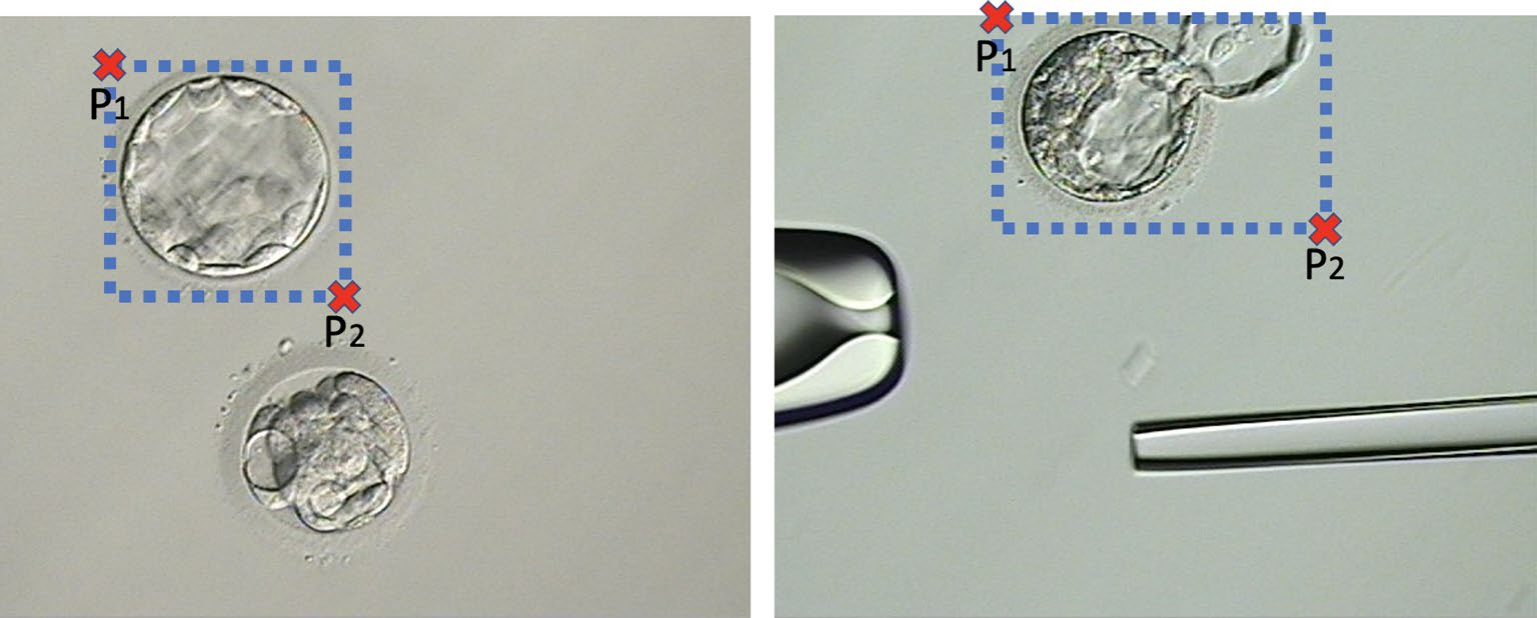
Examples of two blastocyst micrographs and the bounding pox defined by $P_1$ and $P_2$.

For this procedure, we employed a python implementation to detect objects using a deep neural network architecture named“retinanet”^25,26^ (available at: https://github.com/fizyr/keras-retinanet). This network model is trained using a dataset of micrograph images where an expert has manually annotated two points $P_1=(x_1,y_1)$ and $P_2=(x_2,y_2)$ defining the best bounding box that contains the blastocyst of interest (Fig. 5). We employed a transfer learning approach^27^ to train the retinanet, which initializes the weights of the model with those obtained from the training with the ‘imagenet’database^28^. Then the network training was performed using our blastocyst micrograph database for 50 epochs of 1,000 steps each. These parameters were manually set ensuring that the epochs vs. accuracy/loss (learning) graph reach a plateau.

After training, the network model was capable of automatically defining the best bounding box for any input image containing a blastocyst. Using these values, we can automatically crop the original image to obtain an image *O* with the blastocyst centrally located and contained within the minimum area (smallest possible rectangle).

Blastocyst pictures can be captured using a variety of light conditions or magnifications. Therefore, the second part of the pre-processing step consisted of (a) reducing the variation in brightness of the blastocyst regions, and (b) standardizing the images to a common scale.

The reduction of variation in brightness was accomplished by applying a gamma correction algorithm to adjust the overall brightness of an image to avoid regions that are too dark or bright^29^. Then, the images were normalized using a max-min normalization, which adjusts the range of the pixel values from 0 to 255. The gamma correction employed consists of four steps: (i) The median intensity value of all the pixels in the images is computed; (ii) If the median value is between 0.45 and 0.75, the gamma transformation is not performed, otherwise steps (iii) and (iv) are performed until the median value is between 0.45 and 0.75, or three gamma transformations are used; (iii) A gamma transformation is carried out on the pixel values using a gamma value computed using the following function $\gamma = 1.25 * median + 0.375$; and (iv) A new median value is computed from the transformed image and the process is restarted at step (ii).

The standardization of *O* was achieved by applying an image scaling transformation using a bilinear interpolation with a scale factor value $\alpha$. Note that $\alpha$ is defined in such a way that each pixel corresponds to one micrometre according to the magnification factor used when the micrograph was taken.

#### Pixel classification

Let *I* denote the result of pre-processing *O*. The second step consists of generating five binary images so that each pixel of the images indicates its correspondence to each of the regions of interest: zona pellucida $L_{ZP}(x,y)$, trophectoderm $L_{TE}(x,y)$, blastocoel $L_{BC}(x,y)$, inner cell mass $L_{ICM}(x,y)$, and background $L_{BG}(x,y)$. These binary images are generated by employing a neural network classifier that determines the class of each pixel according to a feature vector that describe the image-intensity patterns in its vicinity.

**Figure 6:**
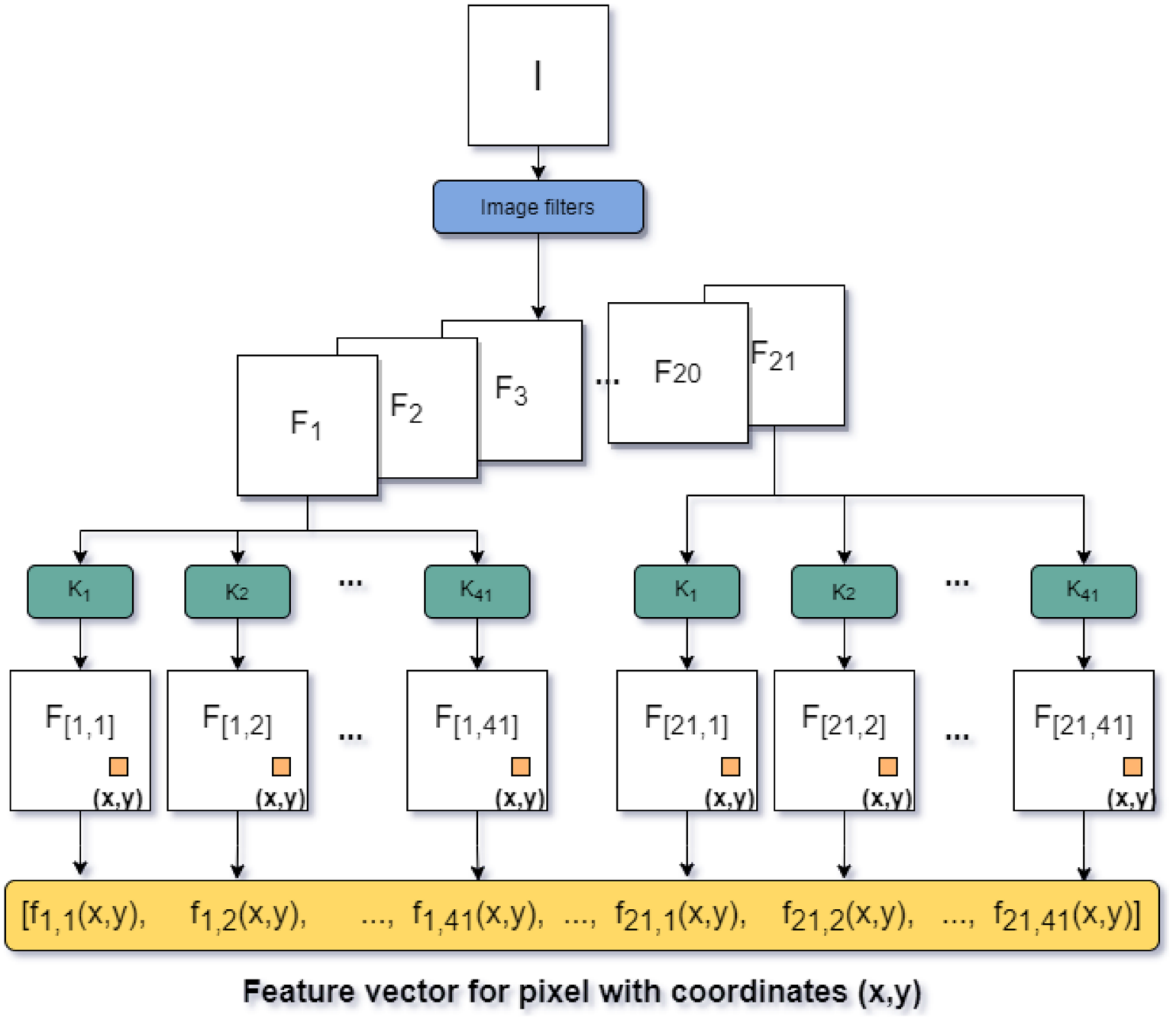
Depiction of the feature extraction process for each pixel. $[F_1,F2,\ldots , F_{21}]$ are filter operations, the symbol $*$ denotes convolution, and $[k_1,k_2,\ldots ,k_41]$ correspond to texture extraction kernels.

The feature vector describing the textural characteristics of each pixel is built by applying 21 filter operations (e.g., entropy, Gaussian blur, Laplacian, and Sobel) on the pre-processed blastocyst micrograph. Then, each of the 21 resulting images is convolved with 41 two-dimensional kernels of size $5 \times 5$ which are designed to extract the texture patterns of patches of different sizes around the centre pixel^30^. As a result, a feature vector of 861 dimensions is generated for each of the pixels in the micrograph. Figure 6 depicts the process to compute the texture feature vector for each pixel of the pre-processed image *I*.

For training the network that classifies each pixel according to its vicinity described above, we randomly selected 50 pixels per blastocyst region of interest for each micrograph, and generated their feature vectors as described previously. This process resulted in a database of 300400 data points that was used to train a neural network (three layers with 400 nodes each, Adam optimizer and categorical cross-entropy as the loss function) during 500 epochs with a validation split of 10% of the training data-size, a callback of learning rate reduction (factor of 0.5, patience of 25 epochs, and validation loss as the monitor) and an early stopping callback (patience of 60 epochs, validation loss as monitor, and a minimal delta of 0.001).

#### Segmentation refinement

**Figure 7:**
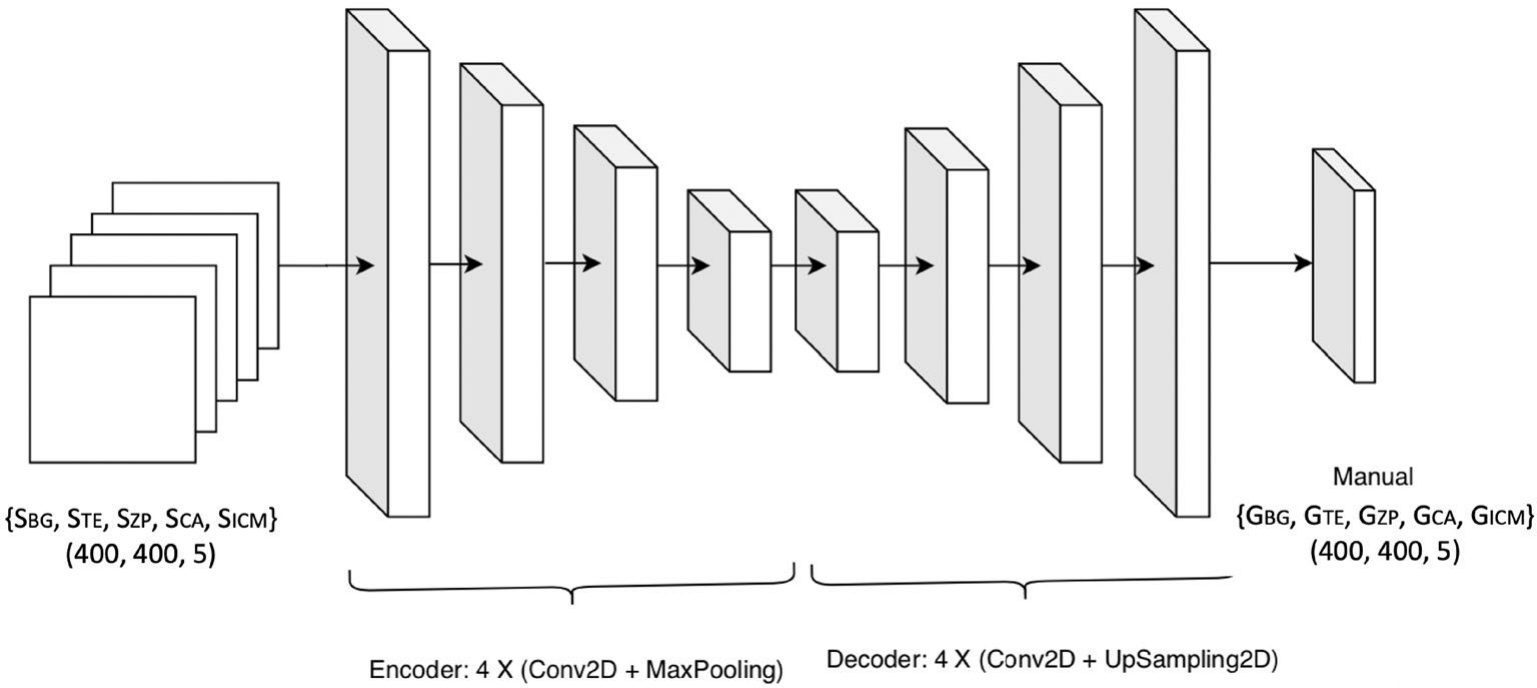
Depiction of the encoder-decoder architecture employed to refine the segmentation result.

Depending on the texture patterns of the blastocyst region, it is possible that the neural network could assign an incorrect class to certain pixels. To improve the results, we incorporated a refinement step to account for the topological characteristics of each region of the embryo. For example, the ZP is the outer layer, and it could be visually (but not structurally) connected to the BG and TE regions, the ICM is contained in the BC, and the TE is located between the ZP and the BC or ICM. To add this knowledge to the proposed method, we employed an encoder-decoder scheme using a deep neural network architecture consisting of three convolutional and three max-pooling layers for the encoding, and four convolutional and three up-sampling layers for the decoding (See Fig. 7). This network is trained using as input a tensor conformed with a concatenation of each of the resulting binary segmentation images *S*. Similarly, a tensor containing the five-ground truth binary masks $G_{BG}$, $G_{TE}$, $G_{ZP}$, $G_{BC}$, and $G_{ICM}$ was set as a target. The proposed model was trained using the mean squared error as the loss function, RMSprop as the optimizer, early stopping, and learning rate reduction call-backs during 100 epochs, with a validation split of 10% of the training data-size. Mean squared error was used since we observed a better performance compared with categorical cross entropy (data not shown).

The validation of the proposed method was measured according to the Dice Similarity Coefficient (DSC)^31^ between the ground truth defined by the manual segmentation images and the segmentation results employing the proposed approach. DSC is a standard metric used to determine the similarity of two segmentation results and is computed as two times the area of overlap divided by the total number of pixels in both images. The value of the DSC indicates overlap between regions, ranging from 0 (indicating no spatial overlap) to 1 (indicating complete overlap).

### Sensitivity analysis and validation

We performed a sensitivity test to evaluate the consistency of the proposed model with respect to changes of location, orientation, and intensity of the input micrographs. For this purpose we used a completely independent dataset of 159 embryo images (122 hatching, 27 expansion, and 10 hatched). The sensitivity was assessed by creating fourteen modified versions of each image using the following parameters.

- Three corresponding to rotations of 90, 180 and 270 degrees.
- Three corresponding to horizontal (H-), vertical (V-), and combined (HV-) flips.
- Eight corresponding to changes in the brightness in the range of [− 100, 100] with increment steps of 25 units.

During our pre-processing procedure, the transformations were performed after the interpolation step, except for the brightness transformation, which was performed after the cropping. The segmentation of each transformed embryo micrograph was compared against the micrograph with no additional transformations, using the DSC.

To test the generalizability and compare against other algorithms, we used the state-of-the-art public repository published by^7^, which was not used at any point during the training process. To pre-process this database of 249 blastocyst micrographs, we assumed that their pixel size was 0.5 micro-meters, since we did not possess that information. Results were compared to those in^7^ using DSC, sensitivity, specificity, precision, and accuracy of the ICM and TE.

## Data Availability

The datasets generated and/or analysed during the current study are not publicly available due they are property of the IVF2.0 Limited company, but are available from the corresponding author on reasonable request.

## References

[1] Zhang JJ (2016). Minimal stimulation IVF vs conventional IVF: A randomized controlled trial. Am. J. Obstet. Gynecol..

[2] Gardner DK, Lane M, Stevens J, Schlenker T, Schoolcraft WB (2000). Blastocyst score affects implantation and pregnancy outcome: Towards a single blastocyst transfer. Fertil. Steril..

[3] Dokras A, Sargent I, Barlow D (1993). Fertilization and early embryology: Human blastocyst grading—an indicator of developmental potential?. Hum. Reprod..

[4] Gardner DK (1999). In-vitro culture of human blastocyst. Towards Reprod. Certain. Infertil. Genet. Beyond.

[5] Chavez-Badiola A, Flores-Saiffe-Farías A, Mendizabal-Ruiz G, Drakeley AJ, Cohen J (2020). Embryo ranking intelligent classification algorithm (erica): Artificial intelligence clinical assistant predicting embryo ploidy and implantation. Reprod. Biomed. Online.

[6] Chavez-Badiola A (2020). predicting pregnancy test results after embryo transfer by image feature extraction and analysis using machine learning. Sci. Rep..

[7] Saeedi P, Yee D, Au J, Havelock J (2017). Automatic identification of human blastocyst components via texture. IEEE Trans. Biomed. Eng..

[8] Rocha JC (2017). A method based on artificial intelligence to fully automatize the evaluation of bovine blastocyst images. Sci. Rep..

[9] VerMilyea M (2020). Development of an artificial intelligence-based assessment model for prediction of embryo viability using static images captured by optical light microscopy during IVF. Hum. Reprod..

[10] Yee, D., Saeedi, P. & Havelock, J. An automatic model-based approach for measuring the zona pellucida thickness in day five human blastocysts. In *Proceedings of the International Conference on Image Processing, Computer Vision, and Pattern Recognition (IPCV)*, 1 (The Steering Committee of The World Congress in Computer Science, Computer 2013).

[11] Rad, R. M., Saeedi, P., Au, J. & Havelock, J. Coarse-to-fine texture analysis for inner cell mass identification in human blastocyst microscopic images. In *2017 Seventh International Conference on Image Processing Theory, Tools and Applications (IPTA)*, 1–5 (IEEE, 2017).

[12] Filho ES (2012). A method for semi-automatic grading of human blastocyst microscope images. Hum. Reprod..

[13] Singh A, Au J, Saeedi P, Havelock J (2014). Automatic segmentation of trophectoderm in microscopic images of human blastocysts. IEEE Trans. Biomed. Eng..

[14] Kheradmand, S., Saeedi, P. & Bajic, I. Human blastocyst segmentation using neural network. In *2016 IEEE Canadian Conference on Electrical and Computer Engineering (CCECE)*, 1–4 (IEEE, 2016).

[15] Rocha, J. C. et al. Using artificial intelligence to improve the evaluation of human blastocyst morphology. In *IJCCI*, 354–359 (2017).

[16] Rad RM, Saeedi P, Au J, Havelock J (2018). Human blastocyst’s zona pellucida segmentation via boosting ensemble of complementary learning. Inform. Med. Unlocked.

[17] Kheradmand, S., Singh, A., Saeedi, P., Au, J. & Havelock, J. Inner cell mass segmentation in human hmc embryo images using fully convolutional network. In *2017 IEEE International Conference on Image Processing (ICIP)*, 1752–1756 (IEEE, 2017).

[18] Rad, R. M., Saeedi, P., Au, J. & Havelock, J. Multi-resolutional ensemble of stacked dilated u-net for inner cell mass segmentation in human embryonic images. In *2018 25th IEEE International Conference on Image Processing (ICIP)*, 3518–3522 (IEEE, 2018).

[19] Harun, M. Y., Huang, T. & Ohta, A. T. Inner cell mass and trophectoderm segmentation in human blastocyst images using deep neural network. In *2019 IEEE 13th International Conference on Nano/Molecular Medicine and Engineering (NANOMED)*, 214–219 (IEEE, 2019).

[20] Harun, M. Y. et al. Image segmentation of zona-ablated human blastocysts. In *2019 IEEE 13th International Conference on Nano/Molecular Medicine and Engineering (NANOMED)*, 208–213 (IEEE, 2019).

[21] Rad, R. M., Saeedi, P., Au, J. & Havelock, J. Blast-net: Semantic segmentation of human blastocyst components via cascaded atrous pyramid and dense progressive upsampling. In *2019 IEEE International Conference on Image Processing (ICIP)*, 1865–1869 (IEEE, 2019).

[22] Rad, R. M., Saeedi, P., Au, J. & Havelock, J. Predicting human embryos’ implantation outcome from a single blastocyst image. In *2019 41st Annual International Conference of the IEEE Engineering in Medicine and Biology Society (EMBC)*, 920–924 (IEEE, 2019). 10.1109/EMBC.2019.8857002 31946044

[23] Rad RM, Saeedi P, Au J, Havelock J (2020). Trophectoderm segmentation in human embryo images via inceptioned u-net. Med. Image Anal..

[24] Bormann CL (2020). Consistency and objectivity of automated embryo assessments using deep neural networks. Fertil. Steril..

[25] Lin, T. -Y., Goyal, P., Girshick, R., He, K. & Dollár, P. Focal loss for dense object detection. In *Proceedings of the IEEE International Conference on Computer Vision*, 2980–2988 (2017).

[26] Lin, T. -Y. et al. Feature pyramid networks for object detection. In *Proceedings of the IEEE Conference on Computer Vision and Pattern Recognition*, 2117–2125 (2017).

[27] Shin H-C (2016). Deep convolutional neural networks for computer-aided detection: CNN architectures, dataset characteristics and transfer learning. IEEE Trans. Med. Imaging.

[28] Deng, J. et al. Imagenet: A large-scale hierarchical image database. In *2009 IEEE Conference on Computer Vision and Pattern Recognition*, 248–255 (IEEE, 2009).

[29] Poynton, C. *Digital Video and HD: Algorithms and Interfaces* (Elsevier, 2012).

[30] Laws, K. I. *Textured image segmentation* (University of Southern California Los Angeles Image Processing INST, Tech. Rep., 1980).

[31] Dice LR (1945). Measures of the amount of ecologic association between species. Ecology.

